# Characterization of tumor-infiltrating lymphocytes and their spatial distribution in triple-negative breast cancer

**DOI:** 10.1186/s13058-024-01932-4

**Published:** 2024-12-06

**Authors:** Eunkyung Han, Hye Yeon Choi, Hyun Jung Kwon, Yul Ri Chung, Hee-Chul Shin, Eun-Kyu Kim, Koung Jin Suh, Se Hyun Kim, Jee Hyun Kim, So Yeon Park

**Affiliations:** 1 Department of Pathology, Seoul National University Bundang Hospital, Seoul National University College of Medicine, Seongnam, Gyeonggi Republic of Korea; 2 Pathology Center, Seegene Medical Foundation, Seoul, Republic of Korea; 3 Department of Surgery, Seoul National University Bundang Hospital, Seoul National University College of Medicine, Seongnam, Gyeonggi Republic of Korea; 4 Division of Hematology and Medical Oncology, Department of Internal Medicine, Seoul National University Bundang Hospital, Seoul National University College of Medicine, Seongnam, Gyeonggi Republic of Korea; 5 Department of Pathology, Seoul National University Bundang Hospital, 82, Gumi-ro 173 Beon-gil, Bundang-gu, Seongnam, Gyeonggi 13620 Republic of Korea

**Keywords:** Tumor infiltrating lymphocyte, Triple-negative breast cancer, Cytotoxic T lymphocyte, NK cell, Spatial distribution

## Abstract

**Background:**

The tumor immune microenvironment, particularly tumor-infiltrating lymphocytes (TILs), plays a critical role in disease progression and treatment response in triple-negative breast cancers (TNBCs). This study was aimed to characterize the composition of TILs and investigate their clinicopathological and prognostic significance with a special focus on the spatial distribution of TILs in TNBCs.

**Methods:**

We analyzed TNBC samples through PanCancer Immune Profiling using NanoString nCounter assays to identify immune-related genes that are expressed differentially in relation to TIL levels and evaluated protein expression of selected markers through immunohistochemical staining on tissue microarrays. For a comprehensive assessment of the expression of cytotoxic T lymphocyte (CTL) and natural killer (NK) cell markers, a CTL-NK score was devised based on CD8^+^, CD56^+^, CD57^+^, GNLY^+^, and GZMB^+^ TIL levels.

**Results:**

Gene expression analysis revealed significant upregulation of CTL and NK cell-associated genes including *GNLY*, *KLRC2*, and *GZMB* in TIL-high TNBCs. Immunohistochemical validation confirmed that TNBCs with higher TILs had a greater amount of CD56^+^, CD57^+^, GNLY^+^, and GZMB^+^ TILs not only in absolute number but also in proportion relative to CD4^+^ or CD8^+^ TILs. High TIL and its subset (CD4^+^, CD8^+^, CD56^+^, CD57^+^, GNLY^+^, and GZMB^+^ TIL) infiltration correlated with favorable clinicopathological features of tumor. In survival analysis, high CTL-NK score was found to be an independent prognostic factor for better disease-free survival (DFS) of the patients. Furthermore, uniformly high TIL infiltration was linked to better DFS, whereas cases with heterogeneous TIL infiltration showed no difference in survival compared to those with uniformly low TIL infiltration.

**Conclusion:**

Our study showed that CTL and NK cell-associated gene expression and protein levels differ significantly according to TIL levels and that CTL-NK score and distribution of TILs within tumors have a prognostic value. These findings emphasize the importance of CTLs and NK cells as well as the spatial uniformity of TIL infiltration in clinical outcome of TNBC patients, providing valuable insights for refining prognostic assessments and guiding immunotherapeutic strategies.

**Supplementary Information:**

The online version contains supplementary material available at 10.1186/s13058-024-01932-4.

## Background

Breast cancer is a heterogeneous group of diseases that exhibit various clinical characteristics, course of disease, and response to treatment; it is broadly categorized into luminal A, luminal B, human epidermal growth factor receptor 2 (HER2)-enriched, and basal-like (or triple-negative) subtypes [1, 2]. Triple-negative breast cancer (TNBC), defined by negativity for estrogen receptor, progesterone receptor, and HER2, is a heterogeneous disease with different molecular characteristics and clinical outcome [3, 4]. Compared to the other subtypes of breast cancer, TNBC exhibits a worse prognosis [2] and has had limited therapeutic options [5].

However, recent advances in immunotherapy for the treatment of TNBC have shown the importance of tumor immune microenvironment (TIME). The TIME, which plays a crucial role in the progression of tumors as well as response to therapies [6], can vary significantly across different breast cancer subtypes [7] and even within TNBCs [8]. It encompasses tumor-infiltrating lymphocytes (TILs) as well as their subtype and activation state, programmed cell death protein 1 (PD-1) and programmed death-ligand 1 (PD-L1)-positive immune cells, macrophages, and myeloid-derived suppressor cells. It also includes immune-related soluble factors such as cytokines and chemokines [6]. Since TNBC has relatively more TILs than the other subtypes [9], it is a primary focus of research on TIME in breast cancer. The role of TILs in prognosis and treatment response varies by subtype [10]. In TNBC, higher TIL infiltration is associated with a better prognosis and a better response to neoadjuvant chemotherapy [10, 11].

TILs identified via hematoxylin and eosin (H&E) staining permit neither a comprehensive assessment of their overall directional impact on tumor suppression/promotion nor an evaluation of their activation status. Therefore, ongoing research continues to focus on studying subtypes of TILs. However, studies related to TIL subsets other than CD4^+^ [12], CD8^+^ [12–14], and FOXP3^+^ T cells [15, 16] are limited; thus, there are needs for a more comprehensive evaluation of the TIME. Detailed profiling of immune-related genes and proteins in TNBC tissues can provide insights into the mechanisms underlying immune responses and tumor progression.

Moreover, the way TIME affects cancer often varies by region [17]. In breast cancer, TIL infiltration is assessed in a representative tumor block according to the guidelines of the International Working Group for TILs (www.tilsinbreastcancer.org). However, the distribution of immune cell infiltration can be uneven within a tumor. A study on TNBC reported that there were transcriptomic differences in immune system pathways between regions with low versus. high TILs via whole RNA sequencing [18]. In addition, another study has revealed that intratumoral spatial heterogeneity of TILs is an important prognostic factor in microsatellite instability-high (MSI-H) colorectal cancer [19]. In breast cancer, however, there is a lack of research on prognostic differences based on spatial heterogeneity of TIL infiltration. Understanding this heterogeneity could potentially refine prognostic stratification and therapeutic decisions.

In this study, we aimed to conduct a comprehensive analysis of TIME in TNBC by comparing high versus low TIL-infiltrated tumors. We investigated the composition of TILs in TNBC and correlated it with clinicopathological features as well as clinical outcomes. In addition, we explored spatial heterogeneity of TIL and its subset infiltration within tumors and their impact on prognosis of TNBC patients.

## Methods

### Tissue samples

All tissue samples used in this study were from patients with primary TNBC who underwent surgical resection at Seoul National University Bundang Hospital. Two sets of tumor samples were used as a discovery set and a validation set, respectively. The first discovery set was composed of 36 cases of TNBC and was used for PanCancer Immune Profiling. To address the issue of RNA quality, the samples were restricted to those which were acquired between 2019 and 2022. They were intentionally matched with regards to recurrence and level of TIL infiltration of a similar number. The baseline clinicopathological characteristics of the 36 cases are presented in Additional file 1: Table S1.

The second validation set was composed of 172 cases of primary TNBC without recurrence at the time of diagnosis that were consecutively resected from 2003 to 2012, and immunohistochemical analyses were performed using tissue microarrays. Of the 172 cases, 17 had received neoadjuvant chemotherapy; these cases were excluded from the analysis because the infiltration of TIL and its subsets was significantly lower compared to those who did not receive neoadjuvant chemotherapy, indicating alteration of the TIME due to treatment. Thus, the final analysis was conducted on 155 cases. The clinicopathological characteristics of the 155 TNBC patients are summarized in Additional file 2: Table S2.

### NanoString nCounter assay

Representative formalin-fixed paraffin-embedded tissue blocks of each tumor were sectioned at a thickness of 8 μm. The sections containing the tumors were marked and dissected, and RNA was extracted using RecoverAll™ Total Nucleic Acid Isolation Kit (Ambion, Grand Island, NY). The isolated RNA was used for the NanoString nCounter multiplex assay (NanoString Technologies, Seattle, WA). PanCancer Immune Profiling Panel for human (NanoString Technologies) was used for this study which targets and quantitates 770 mRNAs, 730 of which are immune-related genes and 40 of which are housekeeping genes that allow sample-to-sample normalization. After hybridization of RNA with the target-specific capture and reporter probes, excess probes were eliminated and the probe/target complexes were then aligned on the nCounter Cartridge (NanoString Technologies). The cartridges were placed into the nCounter Digital Analyzer (NanoString Technologies) for image acquisition and data capture. The automated fluorescence microscope of the analyzer scanned the sample, and the labeled barcodes were directly counted.

### Evaluation of clinicopathological characteristics

Clinicopathological characteristics of the tumors were retrieved from the medical records and from reviewing the H&E or immunohistochemical slides. The following information was recorded: age at diagnosis, sex, histologic type (by WHO classification), TNM stage (by 8th American Joint Committee on Cancer staging system), presence of lymphovascular invasion (LVI), histologic grade including nuclear pleomorphism (by Nottingham histologic grading system), presence of ductal carcinoma in situ (DCIS) component, tumor border (pushing vs. infiltrative), multicentricity, level of TIL infiltration (< 10%, ≥ 10% and < 50%, or ≥ 50%), Ki-67 index, p53 overexpression, and basal phenotype. Basal phenotype was defined as positive membranous staining for either cytokeratin 5/6 or epidermal growth factor receptor. In addition, using the clinical follow-up data in the patients’ medical records, we collected the following information pertinent to survival as well: type or site of recurrence, date of diagnosis for recurrent cancers, date of last contact or death, and type of adjuvant therapy.

### Tissue microarray construction

The tumor section slides were reviewed, and one to two representative slides of each tumor were selected. From these slides, three areas with varying levels of TIL infiltration were chosen. Paired three 2 mm-diameter tissue cores from these areas were collected to create a tissue microarray (TMA) using a trephine apparatus (SuperBioChips Laboratories, Seoul, South Korea).

### Immunohistochemical staining and scoring

Immunohistochemical staining for CD4 (clone SP35; ready to use; Ventana Medical Systems, Tucson, AZ), CD8 (clone C8/144B; ready to use; Dako, Carpinteria, CA), CD56 (clone MRQ-42; ready to use; Ventana Medical Systems), CD57 (clone NK1; ready to use; Abcam, Cambridge, United Kingdom), granulysin (GNLY; polyclonal; 1:200; Abcam), and granzyme B (GZMB; Clone EPR22645-206; 1:500; Abcam) was performed using 4 μm-thick sections of TMA blocks. It was carried out on BenchMark XT autostainer (Ventana Medical Systems) using ultraView Universal DAB Detection Kit (Ventana Medical Systems).

Immunohistochemically-stained TMA slides were scanned using Aperio GT 450 DX (Leica, Wetzlar, Germany), and an open-source digital image analyzer QuPath (version 0.4.3) was used to count TILs expressing each marker. A high-power field (HPF) with the highest number of infiltration was selected in each TMA core, and the numbers of the TIL subsets were counted in each HPF. As a result, counts from the hotspots of three paired cores per case were obtained, and an average value was calculated for each case. The median value of these averages of all 155 cases were used as cut-offs when categorizing the cases into low-expression (≤ median) and high-expression (> median) groups.

Using the average numbers of TIL subset infiltration in each case, the ratios of TIL subsets over CD4^+^ or CD8^+^ TILs were calculated as follows: CD4^+^/CD8^+^, CD56^+^/CD8^+^, CD57^+^/CD8^+^, GNLY^+^/CD8^+^, GZMB^+^/CD8^+^, CD56^+^/CD4^+^, CD57^+^/CD4^+^, GNLY^+^/CD4^+^, and GZMB^+^/CD4^+^ TIL ratio.

For a comprehensive assessment of the expression levels of markers associated with cytotoxic T lymphocyte (CTL) or natural killer (NK) cells, a CTL-NK score was devised. For each five markers (CD8, CD56, CD57, GNLY, and GZMB), a score of 0 was assigned for cases classified into the low-expression group and a score of 1 for those categorized into the high-expression group. These scores were then summed up to derive the final CTL-NK score for each case. Consequently, the score ranged from 0 to 5.

### Evaluation of heterogeneity of TIL infiltration

The overall TIL infiltration was scored using the guidelines of the International Working Group for TILs in breast cancer. Heterogeneity of TIL infiltration was determined by examining the degrees of TIL infiltration in each of the three TMA cores. When it was either low (< 10%) or high (≥ 10%) in all three cores, it was considered to be homogeneously low or high; otherwise, it was described as heterogeneous.

Similarly, evaluation of heterogeneity in TIL subset infiltration was based on whether the extent of infiltration in each of the three hotspots was consistently low (below median) or high (above median). Any instances where there was a mixture of low and high infiltration levels were classified as heterogeneous. When one of the three TMA cores was fragmented or lost during the immunohistochemical staining process and made evaluation of one core impossible, cases were deemed heterogeneous only when TIL subset infiltration showed values below and above the median in two cores, respectively. Cases whose infiltration levels consistently fell below or exceeded the median in both remaining cores were considered inconclusive.

### Statistical analysis

In PanCancer immune profiling using Nanostring nCounter assay, collected data were checked for quality control, normalized, and analyzed by nSolver analysis software (version 4.0) and nCounter Advanced Analysis version 2.0 (NanoString Technologies). R software was used for comparison of the mRNA expression between two groups. Differential gene expression between groups was described with a log2 fold change, and adjusted *p* value using the Benzamini-Yekutieli procedure was presented.

Other statistical analyses were performed using IBM SPSS Statistics for Windows, version 27.0 (IBM Corporation, Armonk, New York). To examine the correlation between TIL, TIL subset, and TIL subset ratio, Spearman’s rank correlation test was employed, and the Rho correlation coefficient was presented. Mann-Whitney U test was used to compare the differences in TIL subset infiltration and TIL subset ratio between low and high TIL groups and to assess the differences in TIL subset infiltration and TIL subset ratio between groups divided by clinicopathological parameters. Comparison of clinicopathological features according to TIL infiltration (low vs. high-TIL) and according to CTL-NK score (< 2 vs. ≥2) was performed by Chi-square test or Fisher’s exact test. Survival curves were plotted by the Kaplan-Meier method, and their significance of differences was assessed using the log-rank test. Corrections for multiple testing were made by Bonferroni method, and adjusted *p* values were calculated. To identify independent factors associated with survival, multivariate analysis using Cox proportional hazard model was conducted with backward stepwise selection model. Hazard ratio (HR) and 95% confidence interval (CI) were calculated for each variable. All *p* values were two-sided, and *p* values less than 0.05 were considered statistically significant.

## Results

### Comprehensive immune profiling using NanoString nCounter assay

**Fig. 1:**
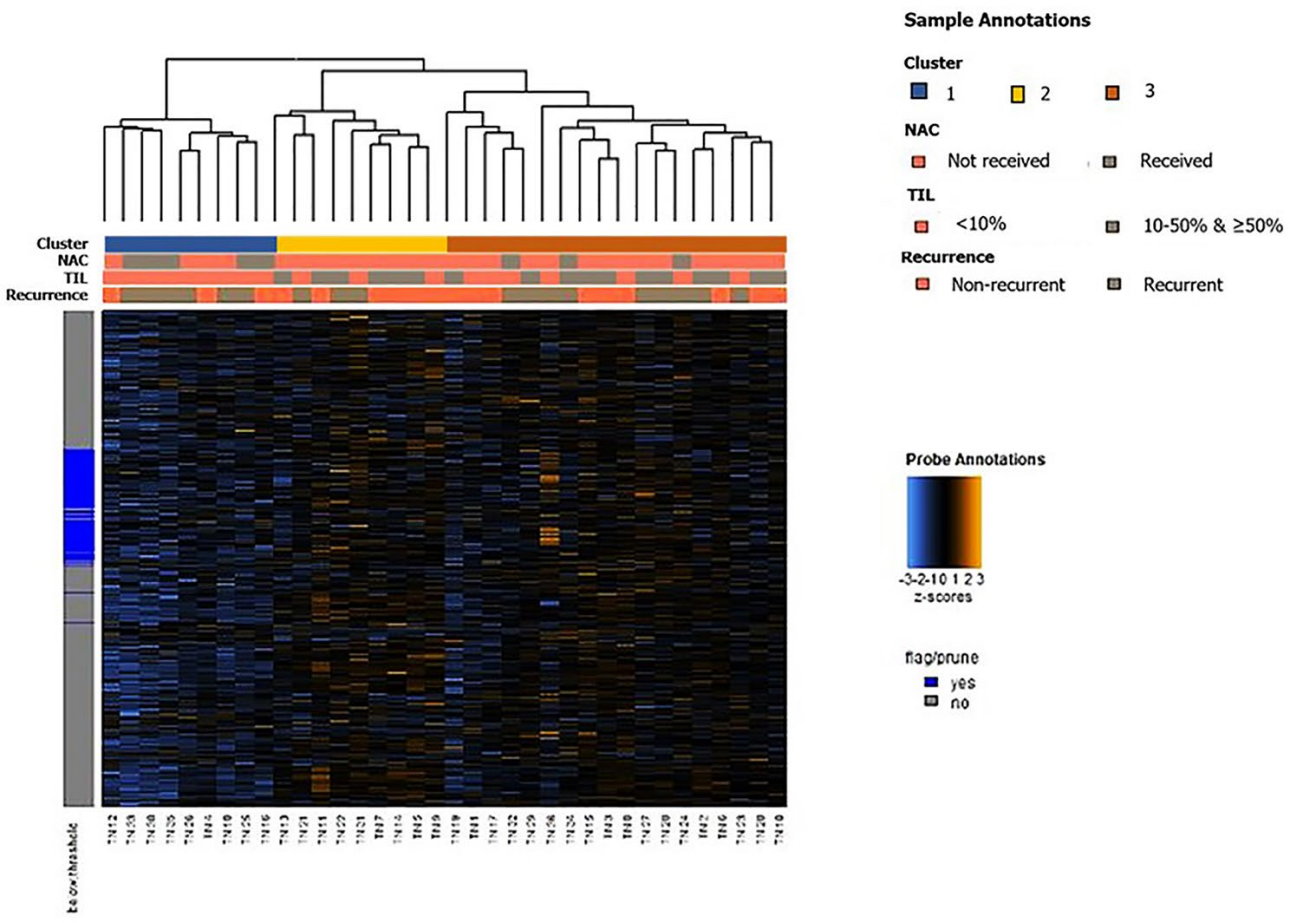
Heatmap of immune-related genes generated by unsupervised clustering. Samples were grouped into three based on their immune-related gene expression; one cluster had lower expression (cluster 1), another one had higher expression (cluster 2), and the last one showed intermediate expression (cluster 3). The clustering of each group was not determined by recurrence status. However, a potential relationship between immune-related gene expression patterns, TIL levels, and prior neoadjuvant chemotherapy (NAC) was observed. Cluster 1 consistently exhibited tumor-infiltrating lymphocyte infiltration of less than 10%. Additionally, none of the cases in Cluster 2 had undergone NAC. Color keys for sample annotation are displayed on the right

As a whole, unsupervised clustering analysis revealed that the samples could be broadly categorized into three clusters based on the overall expression patterns of immune-related genes (Fig. 1): one cluster (cluster 1) characterized by a decrease in the expression of immune-related genes, another cluster (cluster 2) exhibiting an increase in the expression of immune-related genes, and the other cluster (cluster 3) displaying an intermediate level of immune-related gene expression. 

Although the samples were not clustered based on recurrence status, an association between the clusters and the level of TIL infiltration was observed. Cluster 1, which was characterized by a decrease in immune-related gene expression, uniformly exhibited TIL infiltration level of less than 10%. In addition, none of Cluster 2 had undergone neoadjuvant chemotherapy. These findings suggest a potential relationship between the immune-related gene expression patterns, TIL levels, and a history of neoadjuvant chemotherapy within our cohort.

Comparing groups with low (< 10%) and high (≥ 10%) levels of TILs, the top 20 genes exhibiting differential expression are presented in Table 1. Notably, genes showing substantial differences in expression (log2 fold change ≥ 1.5) and statistically-significant adjusted *p* values (< 0.05) included *GNLY*, *KLRC2*, and *GZMB*. This finding suggests that there may be differences in cytotoxicity and the distribution of NK cells between the groups.

**Table 1:** List of top 20 genes with a significant fold change in high-TIL group compared to low-TIL group

Gene	Log2 fold change	*p* value	Adjusted *p* value
GNLY-mRNA	1.78	1.60E-06	0.0063
KLRC2-mRNA	2.22	9.93E-06	0.0170
CD83-mRNA	0.80	1.29E-05	0.0170
TGFB2-mRNA	-1.46	2.25E-05	0.0222
CCL5-mRNA	1.42	3.69E-05	0.0291
CCR6-mRNA	1.38	5.02E-05	0.0318
SELL-mRNA	1.36	5.64E-05	0.0318
IL2RB-mRNA	1.47	6.89E-05	0.0338
ISG20-mRNA	0.99	7.72E-05	0.0338
GZMB-mRNA	1.82	9.45E-05	0.0372
TAP1-mRNA	1.32	0.000183	0.0608
PRF1-mRNA	1.39	0.000185	0.0608
MICB-mRNA	1.03	0.000204	0.0619
CCR7-mRNA	1.61	0.000242	0.0668
PSMB9-mRNA	1.19	0.000254	0.0668
SOCS1-mRNA	1.15	0.000305	0.0704
KLRK1-mRNA	1.33	0.000308	0.0704
NOD2-mRNA	0.90	0.000343	0.0704
CD247-mRNA	1.23	0.000355	0.0704
GZMM-mRNA	1.27	0.000387	0.0704

Log2 fold changes were calculated using tumors with low levels of TIL infiltration as the baseline

*p* values were adjusted by Benzamini-Yekutieli procedure

TIL, tumor-infiltrating lymphocyte

### Evaluation of differentially expressed genes and related TIL subsets

Based on the findings from comprehensive immune profiling of TNBC, we selected immune-related markers GNLY and GZMB for further analysis. Given the association of these markers with CTL and NK cells, CD8^+^, CD56^+^ and CD57^+^ TILs were also evaluated in the validation set. Additionally, CD4^+^ TILs were evaluated to obtain baseline data.

As a whole, TIL infiltration ranged from 1 to 90% with median of 30%. While 48 cases (31.0%) showed less than 10% of TIL infiltration, 107 cases (69.0%) showed greater than or equal to 10% of TIL infiltration. Among the TIL subsets, CD4^+^ and CD8^+^ TILs comprised the majority of the TILs. The median of the average number of infiltrated cells per HPF was 223.7 (range, 0.0-1238.3) for CD4^+^ TIL and 215.0 (range, 0.0-1022.3) for CD8^+^ TIL. On the other hand, CD56^+^, CD57^+^, GNLY^+^, and GZMB^+^ TILs accounted for a smaller portion with the median values of 1.7 (range, 0.0-22.3), 6.7 (0.0-116.7), 13.0 (0.0-313.0), and 7.3 (0.0-244.0), respectively.

When analysis was done based on CD4^+^ or CD8^+^ TILs, the ratios of TIL subsets, expressed as median (inter-quartile range) were as follows: CD4^+^/CD8^+^, 0.93 (0.62–1.42); CD56^+^/CD8^+^, 0.01 (0.00-0.02); CD57^+^/CD8^+^, 0.03 (0.01–0.06); GNLY^+^/CD8^+^, 0.06 (0.02–0.15); GZMB^+^/CD8^+^, 0.03 (0.01–0.14); CD56^+^/CD4^+^, 0.01 (0.00-0.02); CD57^+^/CD4^+^, 0.03 (0.01–0.06); GNLY^+^/CD4^+^, 0.06 (0.03–0.14); GZMB^+^/CD4^+^ TIL ratio, 0.04 (0.01–0.15).

### Correlation between TIL and its subsets infiltration or CTL-NK score

**Fig. 2:**
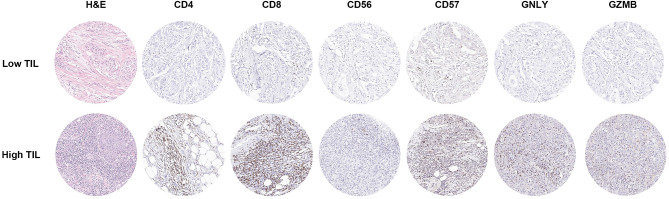
Representative images of H&E and immunohistochemical staining from one case showing low TIL and the other case showing high TIL. The case with low TIL infiltration also shows a low level of CD4^+^, CD8^+^, CD56^+^, CD57^+^, GNLY^+^, and GZMB^+^ cell infiltration. On the other hand, the case with high TIL infiltration shows abundant CD4^+^, CD8^+^, CD56^+^, CD57^+^, GNLY^+^, and GZMB^+^ cell infiltration. Each circle represents one high power field

Next, we examined the correlation between TIL and TIL subset infiltration. Infiltration of TIL and its subsets including CD4^+^, CD8^+^, CD56^+^, CD57^+^, GNLY^+^, and GZMB^+^ TIL showed positive correlations with Rho correlation coefficient ranging from 0.534 to 0.806 (*p* < 0.001; Additional file 3: Table S3). When the infiltration levels of TIL subsets between low-TIL (TIL < 10%) and high-TIL (TIL ≥ 10%) groups were compared, a significant association was also observed for all TIL subsets (*p* < 0.001; Table 2; Fig. 2).

**Table 2:** Comparison of TIL subset and TIL ratio between low-TIL and high-TIL groups

TIL subset or ratio	Low-TIL group (*n* = 48)	High-TIL group (*n* = 107)	*p* value
CD4^+^ TIL	63.7 (31.9-121.2)	288.3 (200.0-427.3)	< 0.001
CD8^+^ TIL	83.3 (43.7-142.3)	279.7 (161.3-386.3)	< 0.001
CD56^+^ TIL	0.0 (0.0-0.7)	3.3 (0.7–7.7)	< 0.001
CD57^+^ TIL	0.7 (0.0-3.2)	10.7 (5.0-18.7)	< 0.001
GNLY^+^ TIL	1.3 (0.1–6.3)	22.7 (11.3–46.0)	< 0.001
GZMB^+^ TIL	1.15 (0.0-5.2)	15.0 (2.3–39.3)	< 0.001
CD4^+^/CD8^+^ TIL	0.64 (0.39–1.18)	0.99 (0.73–1.53)	0.002
CD56^+^/CD8^+^ TIL	0.00 (0.00-0.01)	0.01 (0.00-0.02)	< 0.001
CD57^+^/CD8^+^ TIL	0.01 (0.00-0.03)	0.04 (0.02–0.06)	< 0.001
GNLY^+^/CD8^+^ TIL	0.02 (0.00-0.06)	0.09 (0.04–0.19)	< 0.001
GZMB^+^/CD8^+^ TIL	0.01 (0.00-0.06)	0.06 (0.01–0.16)	0.001
CD56^+^/CD4^+^ TIL	0.00 (0.00-0.01)	0.01 (0.01–0.02)	< 0.001
CD57^+^/CD4^+^ TIL	0.01 (0.00-0.04)	0.04 (0.02–0.07)	< 0.001
GNLY^+^/CD4^+^ TIL	0.03 (0.01–0.07)	0.08 (0.04–0.18)	< 0.001
GZMB^+^/CD4^+^ TIL	0.02 (0.00-0.08)	0.04 (0.01–0.16)	0.029

Data are presented as median (inter-quartile range), and *p* values were calculated by Mann-Whitney U test

TIL, tumor-infiltrating lymphocyte; GNLY, granulysin; GZMB, granzyme B

We also examined the correlation between TIL infiltration and TIL subset ratios. All ratios except for CD4^+^/CD8^+^ TIL ratio showed moderate correlations with TIL infiltration (Rho correlation coefficients of 0.351–0.528; *p* < 0.001; Additional file 4: Table S4). When TIL subset ratios were compared between low-TIL and high-TIL groups, there was a statistically significant difference between the two groups in all TIL subset ratios (Table 2).

As for the CTL-NK score, 60 patients (38.7%) had a CTL-NK score of less than 2, while 95 patients (61.3%) had a score of 2 or higher. The group with a CTL-NK score of less than 2 exhibited a TIL median of 5.0% (inter-quartile range, 5.0-18.8%), whereas those with a CTL-NK score of 2 or higher showed a substantially higher TIL median of 45.0% (inter-quartile range, 20.0–70.0%) (*p* < 0.001).

### Clinicopathological features associated with infiltration of TILs and its subsets

We compared clinicopathological characteristics of low- and high-TIL groups (Additional file 5: Table S5). Notably, the high-TIL group (TIL ≥ 10%) exhibited significant associations with lower T stage (*p* = 0.030), lower rates of LVI (*p* = 0.006), higher nuclear pleomorphism score (*p* = 0.036), absence of DCIS component (*p* = 0.006), and pushing tumor border (*p* = 0.022) compared to the low-TIL group (TIL < 10%). A trend towards higher Ki-67 index (≥ 50%) was observed in the high-TIL group (*p* = 0.051). There were no significant associations between TIL levels and other factors such as age, histologic type, N stage, American Joint Committee on Cancer stage, histologic grade, tumor multicentricity, p53 overexpression, or basal phenotype.

When we examined the relationship between clinicopathological characteristics and TIL subsets (CD8^+^, CD56^+^, CD57^+^, GNLY^+^, GZMB^+^ TIL), we observed similar findings (Table 3). High infiltration of CD8^+^, GNLY^+^, and GZMB^+^ TIL subsets showed a significant association with lower T stage (all *p* < 0.05), and high infiltration of CD56^+^, CD57^+^, GNLY^+^, and GZMB^+^ TIL subset was associated with an absence of LVI (all *p* < 0.05). Especially, high Ki-67 index was associated with higher levels of CD8^+^, CD56^+^, GNLY^+^, and GZMB^+^ TIL infiltration (all *p* < 0.05).

**Table 3:** Relationship between clinicopathological characteristics and CD8^+^, CD56^+^, CD57^+^, GNLY^+^, and GZMB^+^ TIL subsets

Clinicopathological characteristics	CD8^+^ TIL		CD56^+^ TIL		CD57^+^ TIL		GNLY^+^ TIL		GZMB^+^ TIL	
	No. of TILs	*p* value	No. of TILs	*p* value	No. of TILs	*p* value	No. of TILs	*p* value	No. of TILs	*p* value
Age (year)		0.990		0.027		0.306		0.052		0.473
< 50	198.2 (113.7-332.3)		2.3 (0.1–6.9)		7.7 (1.4–15.7)		17.8 (6.1–36.5)		7.7 (1.0–31.0)	
≥ 50	220.3 (80.3-377.3)		0.7 (0.0-4.3)		5.7 (0.7–13.7)		11.3 (2.0–30.0)		5.3 (1.3–22.3)	
Histologic type		0.422		0.755		0.779		0.938		0.979
IC-NST	200.0 (89.1-362.2)		1.7 (0.0–6.0)		6.7 (1.0-15.7)		13.4 (3.3–34.1)		7.5 (1.0-25.5)	
Others^a^	255.3 (144.2-331.9)		1.7 (0.2–6.4)		5.0 (0.7–13.4)		10.7 (1.0-48.4)		7.3 (1.5–31.4)	
T stage		0.044		0.066		0.098		0.005		0.024
T1-2	217.3 (112.4-362.5)		2.0 (0.0–6.0)		6.7 (1.1–15.7)		14.7 (4.3–35.7)		7.7 (1.1–26.0)	
T3-4	70.0 (5.7-256.7)		0.0 (0.0-0.3)		0.7 (0.0-8.7)		1.3 (0.0–7.0)		0.3 (0.0-9.7)	
N stage		0.540		0.343		0.374		0.083		0.125
N0	221.7 (87.2-373.3)		2.0 (0.0–6.0)		6.9 (1.1–15.8)		13.9 (4.7–39.9)		8.5 (1.1–26.0)	
N1-3	188.3 (118.3-294.7)		1.0 (0.0–5.0)		5.7 (1.0-12.3)		12.0 (2.0-19.3)		2.3 (0.3–20.0)	
AJCC stage		0.410		0.087		0.161		0.147		0.030
I-II	219.8 (102.3-365.2)		2.0 (0.0–6.0)		6.9 (1.0-15.9)		14.2 (3.6–36.5)		8.2 (1.3–26.0)	
III	153.3 (115.7-294.7)		0.3 (0.0–4.0)		3.3 (0.5-8.0)		10.7 (2.0-16.7)		1.0 (0.0-7.3)	
LVI		0.169		0.001		0.002		0.001		0.021
Absent	232.3 (117.3–374.0)		2.7 (0.0-7.3)		8.3 (1.3–18.3)		20.3 (4.7–46.0)		9.7 (1.3–31.7)	
Present	170.3 (86.4-315.8)		0.6 (0.0-3.3)		5.0 (0.4–8.5)		11.0 (2.0-19.2)		2.3 (0.4–20.0)	
Histologic grade		0.335		0.066		0.080		0.009		0.041
II	144.7 (67.5-288.5)		0.0 (0.0-3.7)		1.3 (0.2–10.7)		1.3 (0.3–17.2)		1.3 (0.0-4.5)	
III	217.3 (109.3-362.2)		2.0 (0.0–6.0)		6.9 (1.2–15.8)		14.7 (4.7–36.7)		8.2 (1.0–26.0)	
Nuclear pleomorphism		0.104		0.516		0.505		0.032		0.382
2	127.3 (46.0-292.3)		0.0 (0.0-7.3)		7.3 (0.3–11.7)		0.7 (0.3–22.7)		1.7 (0.0-39.3)	
3	219.8 (110.6–357.0)		1.7 (0.0–6.0)		6.5 (1.1–15.6)		14.0 (4.7–35.7)		7.7 (1.0-24.7)	
DCIS component		0.157		0.006		0.066		0.002		0.020
Absent	254.7 (113.8-369.1)		3.0 (0.5–9.2)		8.7 (1.6–18.7)		24.5 (9.6–56.7)		14.5 (2.7–35.3)	
Present	179.3 (103.7-331.3)		1.0 (0.0–5.0)		6.0 (0.7–12.7)		11.3 (2.4–27.7)		3.7 (0.7–22.3)	
Tumor border		0.229		0.038		0.011		< 0.001		0.002
Pushing	234.0 (120.7–366.0)		2.0 (0.0-7.7)		8.0 (2.0–17.0)		21.3 (6.7–46.3)		14.3 (1.0–35.0)	
Infiltrative	185.5 (87.4-319.7)		1.4 (0.0–4.0)		3.7 (0.7–10.3)		6.9 (1.5–17.8)		2.2 (1.0-15.4)	
Multicentricity		0.524		0.951		0.645		0.994		0.911
Absent	217.3 (86.3–347.0)		1.9 (0.0–6.0)		6.7 (1.0-15.7)		13.0 (2.5–36.0)		7.3 (1.0-23.4)	
Present	190.3 (140.3-345.2)		1.0 (0.2–4.2)		6.3 (0.9–13.0)		14.3 (5.8–31.5)		8.7 (1.0-32.5)	
Ki-67 index		0.028		0.011		0.111		0.003		0.015
< 50%	154.6 (77.8–302.0)		0.9 (0.0-4.1)		5.9 (0.7–13.2)		11.0 (1.3–22.7)		2.7 (0.7–20.1)	
≥ 50%	250.3 (122.9-377.8)		3.0 (0.3-7.0)		8.0 (2.0-16.5)		21.3 (6.5–42.7)		14.7 (1.3–36.7)	
p53 overexpression		0.568		0.815		0.629		0.339		0.831
Absent	192.3 (84.5-362.4)		1.7 (0.0–6.0)		6.7 (0.9–16.5)		11.7 (1.3–32.8)		8.7 (0.7–22.4)	
Present	221.7 (109.3-334.5)		1.7 (0.0-5.4)		6.5 (1.2–15.0)		14.5 (5.2–36.0)		6.8 (1.2–26.6)	
Basal phenotype		0.385		0.515		0.397		0.844		0.552
Absent	155.5 (86.3-292.3)		2.0 (0.0–7.0)		7.0 (0.3–13.0)		16.7 (0.7–34.3)		7.3 (0.5–22.7)	
Present	219.8 (107.6–357.0)		1.7 (0.0-5.3)		6.5 (1.1–15.6)		13.0 (4.3–35.3)		7.5 (1.0-27.7)	

Data are presented as median (inter-quartile range), and *p* values were calculated by Mann-Whitney U test

^a^Include metaplastic carcinoma and carcinoma with apocrine differentiation

TIL, tumor-infiltrating lymphocyte; GNLY, granulysin; GZMB, granzyme B; IC-NST, invasive carcinoma of no special type; AJCC, American Joint Committee on Cancer; LVI, lymphovascular invasion; DCIS, ductal carcinoma in situ

Analysis of the relationship between the clinicopathological features and the TIL subset ratios (CD4^+^/CD8^+^, CD56^+^/CD8^+^, CD57^+^/CD8^+^, GNLY^+^/CD8^+^, GZMB^+^/CD8^+^, CD56^+^/CD4^+^, CD57^+^/CD4^+^, GNLY^+^/CD4^+^ and GZMB^+^/CD4^+^ TIL ratio) also yielded similar results (Additional file 6 and 7: Tables S6 and S7).

### Impact of TIL, TIL subsets, and CTL-NK score on patient survival

**Fig. 3:**
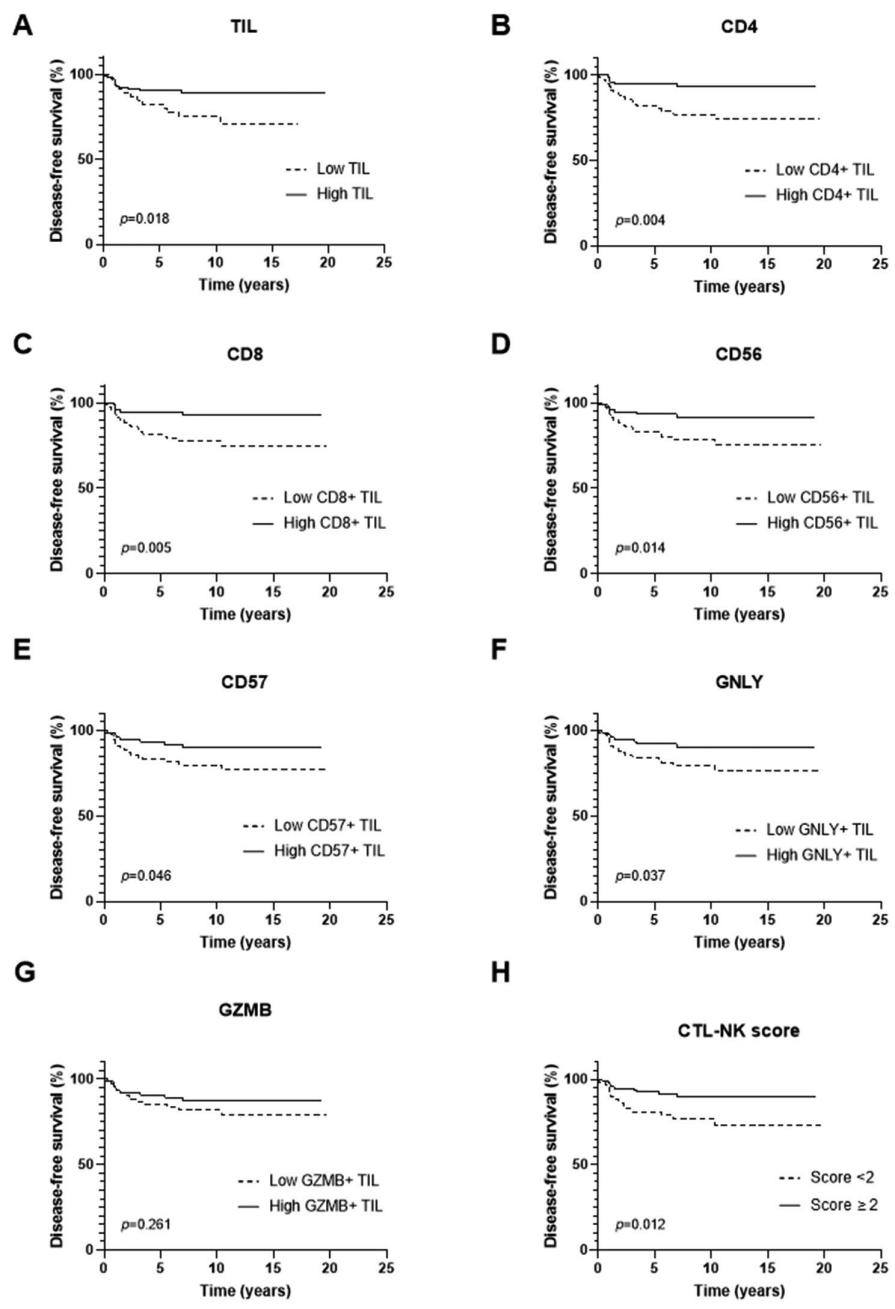
Kaplan-Meier survival curves for disease-free survival according to TIL, TIL subsets, and CTL-NK score. (**A**-**F**) High infiltration of TIL and CD4^+^, CD8^+^, CD56^+^, CD57^+^ and GNLY^+^ TIL subset was associated with increased disease-free survival (*p* = 0.018, 0.004, 0.005, 0.014, 0.046, and 0.037, respectively). **(G)** GZMB^+^ TIL subset infiltration was not associated with disease-free survival (*p* = 0.261). **(H)** CTL-NK score of 2 or higher was associated with increased disease-free survival (*p* = 0.012)

Survival analysis was conducted in the validation set composed of 155 patients. The median follow-up period was 10.04 years (range, 0.17–19.68). During this period, there were 23 recurrences and 16 deaths. The Kaplan-Meier survival curves for disease-free survival (DFS) are shown in Fig. 3. Higher infiltration of TILs and TIL subset (CD4^+^, CD8^+^, CD56^+^, CD57^+^, and GNLY^+^ TIL) along with CTL-NK score of 2 or higher were associated with better DFS of patients (all *p* < 0.05 by log-rank test) while GZMB^+^ TIL did not show a significant association (*p* = 0.261).

Next, we conducted survival analyses by combining TIL levels and CTL-NK scores in order to investigate whether DFS differed depending on CTL-NK score (< 2 vs. ≥2) even within the patients with similar TIL levels. The patients were divided into four groups: TIL-low and CTL-NK score-low, TIL-low and CTL-NK score-high, TIL-high and CTL-NK score-low, and TIL-high and CTL-NK score-high. For these analyses, TIL levels were divided using cutoffs of 10% and 30% (median of TIL levels). Although statistically significant differences were not found among the four groups (*p* = 0.054 for TIL 10% cutoff; *p* = 0.089 for TIL 30% cutoff; Additional file 8: Figure S1), there were some trends of survival differences between two groups. TIL-high and CTL-NK score-high group tended to have better DFS than TIL-high and CTL-NK score-low group (*p* = 0.106 for TIL 10% cutoff, *p* = 0.102 for TIL 30% cutoff).

In addition, the impacts of clinicopathological features, TIL, TIL subsets, and CTL-NK score on the DFS and overall survival (OS) were collectively analyzed using univariate Cox proportional hazard model (Table 4). The clinicopathological variables associated with DFS included T stage (*p* = 0.049) and LVI (*p* = 0.003). TIL, its subsets (except for CD57^+^ and GZMB^+^ TIL), and CTL-NK score showed a significant association with DFS (all *p* < 0.05). However, none of TIL, TIL subsets, or CTL-NK score showed a significant association with OS of patients.

**Table 4:** Univariate analyses of disease-free survival and overall survival

Variables	Category	Disease-free survival		Overall survival	
		HR (95% CI)	*p* value	HR (95% CI)	*p* value
Age	< 50 yrs vs. ≥50 yrs	0.775 (0.335–1.792)	0.552	0.964 (0.359–2.588)	0.941
Histologic type	IC-NST vs. others^a^	1.609 (0.478–5.416)	0.443	0.697 (0.092–5.281)	0.727
T stage	T1-2 vs. T3-4	3.386 (1.003–11.436)	0.049	1.513 (0.199–11.500)	0.689
N stage	N0 vs. N1-3	1.935 (0.837–4.473)	0.123	1.759 (0.639–4.843)	0.274
LVI	Absent vs. Present	3.643 (1.544–8.595)	0.003	3.054 (1.109–8.407)	0.031
Histologic grade	II vs. III	1.015 (0.238–4.329)	0.984	1.494 (0.197–11.316)	0.697
DCIS	Absent vs. Present	3.005 (0.892–10.120)	0.076	1.395 (0.449–4.338)	0.565
Tumor border	Pushing vs. Infiltrative	0.885 (0.375–2.089)	0.781	1.740 (0.652–4.645)	0.269
Multicentricity	Absent vs. Present	1.482 (0.550–3.993)	0.437	1.718 (0.554–5.328)	0.349
TIL	< 10% vs. ≥10%	0.387 (0.171–0.878)	0.023	0.693 (0.252–1.911)	0.479
CD4^+^ TIL	Low vs. High	0.258 (0.096–0.696)	0.007	0.439 (0.152–1.263)	0.127
CD8^+^ TIL	Low vs. High	0.266 (0.099–0.716)	0.009	0.587 (0.213–1.617)	0.303
CD56^+^ TIL	Low vs. High	0.328 (0.129–0.833)	0.019	0.578 (0.210–1.592)	0.289
CD57^+^ TIL	Low vs. High	0.416 (0.171–1.012)	0.053	0.593 (0.215–1.631)	0.311
GNLY^+^ TIL	Low vs. High	0.401 (0.165–0.976)	0.044	0.563 (0.204–1.551)	0.266
GZMB^+^ TIL	Low vs. High	0.621 (0.269–1.436)	0.266	0.985 (0.370–2.625)	0.976
CTL-NK score	0–1 vs. 2–5	0.357 (0.154–0.825)	0.016	0.451 (0.168–1.212)	0.114

*p* values were calculated by Cox proportional hazards model

^a^Include metaplastic carcinoma and carcinoma with apocrine differentiation

HR, hazard ratio; CI, confidence interval; Yrs, years; IC-NST, invasive carcinoma of no special type; LVI, lymphovascular invasion; DCIS, ductal carcinoma in situ; TIL, tumor-infiltrating lymphocyte; DCIS, ductal carcinoma in situ; GNLY, granulysin; GZMB, granzyme B; CTL, cytotoxic T lymphocyte; NK, natural killer

As factors such as TIL, its subsets, and CTL-NK score showed moderate to strong correlations with each other (ρ = 0.534–0.853, *p* < 0.001), multivariate analyses were conducted using different models (Table 5). Across various models, LVI was consistently found to be an independent prognostic factor for worse DFS. Additionally, specific subsets of TILs, notably CD4^+^ (HR, 0.307; 95% CI, 0.112–0.841; *p* = 0.022) and CD8^+^ TILs (HR, 0.312; 95% CI, 0.115–0.844; *p* = 0.022), and the CTL-NK score (HR, 0.420; 95% CI, 0.181–0.978; *p* = 0.044) were revealed as independent prognostic factors of better DFS.

**Table 5:** Multivariate analyses of disease-free survival

Model	Variables	Category	Multivariate analysis	
			HR (95% CI)	*p* value
A	T stage	T1-2 vs. T3-4	2.008 (0.573–7.036)	0.276
	N stage	N0 vs. N1-3	1.129 (0.449–2.842)	0.796
	LVI	Absent vs. Present	2.784 (1.050–7.385)	0.040
	TIL	< 10% vs. ≥10%	0.546 (1.050–7.385)	0.163
B	T stage	T1-2 vs. T3-4	1.677 (0.480–5.859)	0.418
	N stage	N0 vs. N1-3	1.007 (0.395–2.563)	0.989
	LVI	Absent vs. Present	3.055 (1.160–8.041)	0.024
	CD4^+^ TIL	Low vs. High	0.307 (0.112–0.841)	0.022
C	T stage	T1-2 vs. T3-4	2.170 (0.624–7.549)	0.223
	N stage	N0 vs. N1-3	1.143 (0.456–2.869)	0.776
	LVI	Absent vs. Present	2.822 (1.086–7.331)	0.033
	CD8^+^ TIL	Low vs. High	0.312 (0.115–0.844)	0.022
D	T stage	T1-2 vs. T3-4	2.012 (0.570–7.094)	0.277
	N stage	N0 vs. N1-3	1.140 (0.441–2.952)	0.787
	LVI	Absent vs. Present	2.676 (0.985–7.275)	0.054
	CD56^+^ TIL	Low vs. High	0.441 (0.169–1.150)	0.094
E	T stage	T1-2 vs. T3-4	2.486 (0.718–8.602)	0.150
	N stage	N0 vs. N1-3	1.210 (0.483–3.029)	0.684
	LVI	Absent vs. Present	2.853 (1.098–7.413)	0.031
	CD57^+^ TIL	Low vs. High	0.462 (0.188–1.132)	0.091
F	T stage	T1-2 vs. T3-4	1.752 (0.489–6.275)	0.389
	N stage	N0 vs. N1-3	1.211 (0.486–3.021)	0.681
	LVI	Absent vs. Present	2.838 (1.085–7.423)	0.034
	GNLY^+^ TIL	Low vs. High	0.525 (0.207–1.332)	0.175
G	T stage	T1-2 vs. T3-4	2.239 (0.651–7.709)	0.201
	N stage	N0 vs. N1-3	1.173 (0.46–2.957)	0.735
	LVI	Absent vs. Present	2.829 (1.081-7.400)	0.034
	CTL-NK score	0–1 vs. 2–5	0.420 (0.181–0.978)	0.044

*p* values were calculated by Cox proportional hazards model using the backward stepwise selection method

HR, hazard ratio; CI, confidence interval; TIL, tumor-infiltrating lymphocyte; GNLY, granulysin; GZMB, granzyme B

### Heterogeneity of TIL and its subset infiltration and their impact on patient survival

**Fig. 4:**
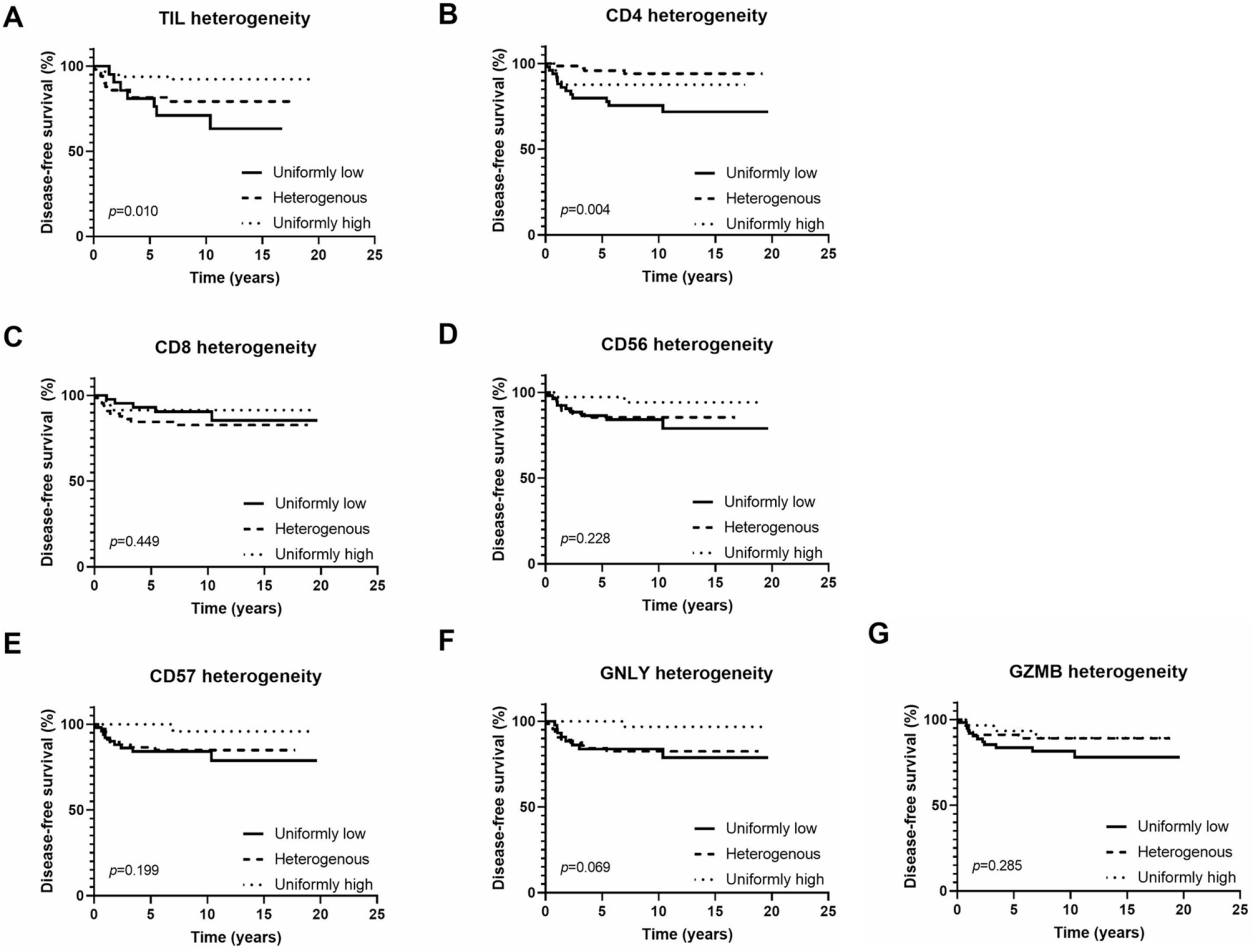
Kaplan-Meier survival curves for disease-free survival according to heterogeneity in TIL infiltration and its subset infiltration. **(A)** A significant difference in disease-free survival was noted between the group with uniformly high infiltration and the group with uniformly low infiltration as well as between the group with heterogeneous infiltration and the group exhibiting uniformly high infiltration (adjusted *p* = 0.009 for both). **(B)** Regarding CD4^+^ TIL subset infiltration, better prognosis was observed in groups with heterogeneous levels compared to those with consistently low levels (adjusted *p* = 0.003). **(C)** When disease-free survival was compared among the groups with different levels of heterogeneity of CD8^+^ TIL subset infiltration, no significant differences were observed between the groups. **(D-F)** Although not statistically significant, similar trends for poor survival were noted in both uniformly low and heterogeneous infiltration groups of CD56^+^, CD57^+^, and GNLY^+^ TIL subset. **(G)** When the disease-free survival was assessed among the groups divided based on the heterogeneity of GZMB^+^ TIL subset infiltration, no significant differences were observed among them

Heterogeneity in TIL levels was observed in 32.3% of cases while heterogeneity in the infiltration of CD4^+^, CD8^+^, C56^+^, CD57^+^, GNLY^+^, and GZMB^+^ TIL was observed in 47.7%, 43.2%, 36.8%, 44.5%, 41.9%, and 36.8% of cases, respectively (Additional file 9: Table S8). Figure 4 shows DFS of different groups based on heterogeneity in TILs and their subset infiltration levels. When cases were divided into three groups based on the heterogeneity of TIL infiltration, a significant difference in DFS was observed among the groups (*p* = 0.010). Groups with heterogeneous TIL levels seemed to exhibit survival intermediate between uniformly high and low groups. However, post-hoc analysis revealed no significant difference between the group showing uniformly low infiltration and the group showing heterogeneous infiltration (adjusted *p* = 1.125). Statistically significant differences were observed between the group with uniformly high infiltration and the group with uniformly low infiltration as well as between the group showing heterogeneous infiltration and the group showing uniformly high infiltration (adjusted *p* = 0.009 for both).

A significant difference in DFS was also observed between the groups based on heterogeneity in CD4^+^ TIL infiltration (*p* = 0.004). When a post-hoc analysis was conducted, a difference in survival was observed only between tumors with uniformly low infiltration and those with heterogeneous infiltration (adjusted *p* = 0.003). Although not statistically significant, for CD56^+^, CD57^+^, and GNLY^+^ TILs, tumors with uniformly low levels of infiltration and those with heterogeneous levels of infiltration tended to exhibit a similar degree of poor prognosis.

## Discussion

This study provides an in-depth characterization of TILs and their clinicopathological and prognostic significance in TNBC with a particular focus on CTLs and NK cells as well as their spatial distributions. We employed the NanoString nCounter PanCancer Immune Profiling Panel to assess the expression of immune-related genes in primary TNBC samples. Comparing TNBC with low TIL levels to those with high TIL levels revealed significantly different expression of the genes *GNLY*,* KLRC2*, and *GZMB*, which encode proteins GNLY, killer cell lectin-like receptor subfamily C member 2 (KLRC2), and GZMB, respectively. GNLY is a pore-forming protein found in the cytotoxic granules of human killer cells such as CTLs and NK cells. Human GNLY is initially present as a 15 kDa protein which is later cleaved to generate a 9 kDa molecule [20], which enables cytotoxic immune cells to directly induce cell death including tumor cells [21]. On the other hand, the 15 kDa form promotes chemo-attraction of additional immune cell populations and maturation of antigen-presenting cells [22]. GZMB is the most prevalent serine protease found in the secretory granules of CTLs and NK cells. GZMB-induced cell death is a key mechanism by which CTLs and NK cells eliminate harmful cells including allogeneic, virus-infected, and tumor cells [23]. In addition, GZMB directly modulates inflammation by enhancing the pro-inflammatory activity of cytokines such as interleukin-1 (IL-1) and interleukin-18 (IL-18) [24, 25]. NK cells have receptors for major histocompatibility complex (MHC) class I molecules that regulate cell-mediated cytotoxicity and cytokine production [26]. One such receptor, encoded by the *KLRC2* gene, is KLRC2, also known as NK cell Group 2 isoform C (NKG2C). NKG2 receptors are heterodimers expressed on NK cells and a small subset of T cells with their function dependent on the isoform. Specifically, NKG2C expression leads to cell activation and enhances cytotoxic function [27]. Therefore, from the different expression of *GNLY*, *KLRC2*, and *GZMB* genes in TNBC with low TIL levels and those with high TIL levels, it could be inferred that NK cells and CTLs are more numerous or more active in TNBCs with high TILs.

Next, we validated the expression of selected immune-related markers through immunohistochemical staining in a larger cohort of TNBC cases. Since KLRC2 expression in tissue has not been confirmed for immunohistochemistry, immunohistochemical staining was performed only for GNLY and GZMB. As mentioned above, it was notable that all three of these genes encoded proteins that are expressed in CTLs and NK cells. Therefore, in order to determine whether a high expression of these genes is associated with abundance of cytotoxic TIL subsets in TNBC with high TIL levels, we performed immunohistochemistry for CD8, CD56, and CD57, the latter two of which were selected as markers to identify NK cells. By definition, NK cells are identified by the presence of CD56 and/or CD16 markers and the lack of the CD3 marker found on T cells [28]. CD57 is recognized as a marker of terminal differentiation with CD57^+^ NK cells exhibiting reduced proliferation yet heightened cytotoxicity against tumor cells compared to CD57^−^ NK cells [29]. Lastly, as CD4^+^ T cells are known to constitute a substantial portion of TILs in invasive breast carcinoma after CD8^+^ T cells [30], we performed immunohistochemistry for CD4 and CD8 as a baseline comparison. During TMA validation, CD4^+^, CD8^+^, CD56^+^, CD57^+^, GZMB^+^, and GNLY^+^ TILs exhibited a significant positive correlation with overall TIL levels. In addition, when we calculated the TIL subset ratios of CD56^+^, CD57^+^, GZMB^+^, and GNLY^+^ TILs using CD4^+^ and CD8^+^ TILs as denominators, a correlation with overall TIL levels was also observed. These findings suggest that as TILs increase, the numbers of NK cells and cytotoxic molecules increase more significantly compared to CD4^+^ and CD8^+^ T cells. These findings collectively suggest that CTLs and NK cells may play a crucial role in TNBC with high TIL levels. NK cells are innate immune effectors exhibiting natural cytotoxicity against primary tumor cells and also play a role in immune-surveillance of metastasis [31, 32]. In addition to their cytotoxic functions, NK cells secrete numerous cytokines, primarily interferon-γ (IFN-γ), which modulate adaptive immune responses [33]. Using the validation set, we demonstrated that TNBC with high TIL levels also has a high number of CTLs and NK cells. Considering that they have antitumor functions, it may be inferred that TNBC with high TIL levels have an association with prolonged survival or favorable prognostic features as discussed below.

High-TIL TNBC was associated with several favorable clinicopathological features including lower T stage and absence of LVI. Similarly, TIL subsets including CD8^+^, CD56^+^, CD57^+^, GNLY^+^, and GZMB^+^ TIL showed a significant association with lower T stage or absence of LVI. These correlations suggest a possibility that a robust immune response, as evidenced by high TIL levels or high CTL and NK cell infiltration, may inhibit tumor progression. This finding is in line with a study by Burnstein et al. [3] who showed that the basal-like immune-suppressed subtype of TNBC, which shows downregulation of B cell, T cell, and NK cell immune-regulating and cytokine pathways, had the least favorable prognosis while the basal-like immune-activated subtype, which exhibits upregulation of immune-related genes, had the best prognosis. However, it is also possible that tumor progression leads to a decrease in TIL levels. A recent study on TNBC showed that during metastasis, there is a general decrease in immune-related gene expression and a reduction in stromal TILs [34]. Another study using flow cytometry revealed that NK cell activity in breast cancer decreases with advancing cancer stage [35]. Thus, it seems that the TIME of TNBC shifts towards an immune-suppressed state as the disease progresses.

In order to evaluate the degree of infiltration of CTL and NK cells comprehensively, we devised a CTL-NK score based on the expression levels of CD8^+^, CD56^+^, CD57^+^, GNLY^+^, and GZMB^+^ TILs. The fact that this score was shown to be a significant independent prognostic factor for improved DFS in the current study indicates the prognostic value of combined CTL and NK cell infiltration. CD8^+^ T cells have been widely reported to be associated with a favorable prognosis in TNBC [10, 11], and NK cells are reported to be linked to a favorable prognosis in many solid tumors [36] including colorectal cancer [37], gastric cancer [38], lung cancer [39], renal cell carcinoma [40], and melanoma [41]. One meta-analysis found that high levels of NK cell markers CD56 and CD57 in solid tumor tissues could predict favorable prognosis and serve as an independent prognostic marker for favorable OS [42]. In breast cancer, one study found that high NK cell infiltration was significantly associated with TILs and that high CD56 expression (≥ 5 cells/10 HPFs) correlated with improved OS and DFS [43]. GNLY and GZMB have been reported to be associated with a better prognosis in solid tumors as well. One study showed that upregulated mRNA expression of intratumoral GNLY and GZMB was associated with a favorable outcome in patients with colorectal cancer [44]. Another study on primary untreated breast cancer patients revealed that high levels of *GNLY* mRNA predict a low risk of local recurrence but a high risk of distant metastasis [45]. Increased numbers of GZMB^+^ cells in the TIME have been linked to a favorable prognosis in some tumors including cervical intraepithelial neoplasia [46], oral squamous cell carcinoma [47], and melanoma [48]. Our findings that are consistent with the results of previous studies emphasize the need for an integrated assessment of multiple immune cell types in TNBC beyond the traditional focus on overall TILs.

The spatial heterogeneity of TIL infiltration within tumors was also found to be a crucial factor associated with patient prognosis. Uniformly high TIL infiltration was associated with better DFS while heterogeneous TIL infiltration did not confer the same survival benefit. This is a significant finding since it suggests that in addition to the quantity of TILs, the distribution of TILs within the TIME impacts clinical outcomes. This is in line with a previous study on MSI-H colon cancer where TIL-low/heterogeneity-high, TIL-low/heterogeneity-low, and TIL-high subgroups were significantly associated with poor, intermediate, and good DFS [19].

There are some limitations in this study. First, the relatively small sample size, particularly in the discovery set, may limit generalization of the results. Further research is needed to confirm the findings of our study in a large cohort. Secondly, the discovery set included some post-neoadjuvant chemotherapy samples which showed significantly lower TIL and its subset infiltration compared to the non-treated samples, whereas the final validation set was only composed of non-treated samples. This may lead to a bias in the results. Thirdly, although we selected representative tumor areas to construct TMAs, they may not reflect the TIME of the entire tumor due to a sampling issue. Furthermore, we selected a hotspot in a TMA core to evaluate TIL subset infiltration. Scanning of the whole slide of the tumor with subsequent digital analyses of spatial heterogeneity of TIL and its subset infiltration may be a next step to extend this study.

## Conclusion

In summary, this study showed that CTL and NK cell-associated gene expression and protein levels differ significantly according to TIL levels in TNBCs, with higher CTLs and NK cells in the high-TIL group. We developed a CTL-NK score which reflects overall CTL and NK cell infiltration that also has a prognostic value in TNBCs. More importantly, our study showed that spatial distribution of TILs within a tumor has a prognostic implication. While tumors with homogeneously high TIL infiltration showed favorable clinical outcome, those with heterogeneous TIL infiltration was associated with poor clinical outcomes similar to those with homogeneously low TIL infiltration. Collectively, this study demonstrates the importance of NK cells as well as CTLs and the spatial distribution of TILs in prognosticating TNBC patients, providing valuable insights into understanding the TIME as well as potential therapeutic strategies in TNBCs.

## Electronic supplementary material

Below is the link to the electronic supplementary material.

Supplementary Material 1: Additional file 1: Table S1. Clinicopathological characteristics of 36 TNBC patients in the test set. Additional file 2: Table S2. Clinicopathological characteristics of 155 TNBC patients in the validation set. Additional file 3: Table S3. Correlation between tumor infiltrating lymphocyte and its subsets. Additional file 4: Table S4. Correlation between tumor infiltrating lymphocyte and its subset ratios. Additional file 5: Table S5. Relationships between TIL levels and clinicopathological features of tumors. Additional file 6: Table S6. Relationship between clinicopathological characteristics and TIL subset ratios using CD8^+^ TIL. Additional file 7: Table S7. Relationship between clinicopathological characteristics and TIL subset ratios using CD4 + TIL. Additional file 9: Table S8. Distribution of tumor infiltrating lymphocyte and its subsets.

Supplementary Material 2: Additional file 8: Figure S1. Kaplan-Meier survival curves for disease-free survival according to combined analyses of TIL levels (A, < 10% or ≥ 10%; B, < 30% or ≥ 30%) and CTL-NK scores (< 2 or ≥ 2).

## Data Availability

No datasets were generated or analysed during the current study.

## Abbreviations

CTL: Cytotoxic T lymphocyte
DCIS: Ductal carcinoma in situ
DFS: Disease-free survival
GNLY: Granulysin
GZMB: Granzyme B
H&E: Hematoxylin and eosin
HPF: High-power field
HER2: Human epidermal growth factor receptor 2
KLRC2: Killer cell lectin-like subfamily C member 2
LVI: Lymphovascular invasion
MSI-H: Microsatellite instability-high
NK cell: Natural killer cell
NKG2C: NK cell Group 2 isoform C
TIL: Tumor-infiltrating lymphocyte
TIME: Tumor immune microenvironment
TMA: Tissue microarray
TNBC: Triple-negative breast cancer
OS: Overall survival
