# Supplementary material for: Characterization of tumor-infiltrating lymphocytes and their spatial distribution in triple-negative breast cancer
Source: Breast Cancer Res. 2024 Dec 6;26:180. doi: 10.1186/s13058-024-01932-4 (PMC11622547; doi:10.1186/s13058-024-01932-4)
Supplement: Supplementary file 1 — Supplementary Material 1: Additional file 1: Table S1. Clinicopathological characteristics of 36 TNBC patients in the test set. Additional file 2: Table S2. Clinicopathological characteristics of 155 TNBC patients in the validation set. Additional file 3: Table S3. Correlation between tumor infiltrating lymphocyte and its subsets. Additional file 4: Table S4. Correlation between tumor infiltrating lymphocyte and its subset ratios. Additional file 5: Table S5. Relationships between TIL levels and clinicopathological features of tumors. Additional file 6: Table S6. Relationship between clinicopathological characteristics and TIL subset ratios using CD8+ TIL. Additional file 7: Table S7. Relationship between clinicopathological characteristics and TIL subset ratios using CD4 + TIL. Additional file 9: Table S8. Distribution of tumor infiltrating lymphocyte and its subsets. [file 13058_2024_1932_MOESM1_ESM.pdf]

Table S1. Clinicopathological characteristics of 36 TNBC patients in the test set

Characteristics	No (%)
Age at primary diagnosis, years	
<50	17 (47.2)
≥50	19 (52.8)
Sex	
Female	36 (100.0)
Male	0 (0.0)
Histologic type	
Invasive carcinoma of no special type	31 (86.1)
Metaplastic carcinoma	4 (11.1)
Carcinoma with apocrine differentiation	1 (2.8)
Neoadjuvant chemotherapy	
Not received	28 (77.8)
Received	8 (22.2)
Disease progression	
Not recurred	18 (50.0%)
Recurred	18 (50.0%)
T stage	
1	13 (36.1)
2	15 (41.7)
3	7 (19.4)
4	1 (2.8)
N stage	
0	21 (58.3)
1	6 (16.7)
2	4 (11.1)
3	5 (13.9)
Lymphovascular invasion	
Absent	22 (61.1)
Present	14 (38.9)
Histologic grade	
I	0 (0.0)
II	2 (5.6)
III	34 (94.4)
DCIS component	
Absent	10 (27.8)
Present	26 (72.2)
Multicentricity	
Absent	27 (75.0)
Present	9 (25.0)
Tumor-infiltrating lymphocyte	
<10%	19 (52.8)
≥10% and <50%	15 (41.7)
≥50%	2 (5.6)
Ki-67 index	
<50%	16 (44.4)
≥50%	20 (55.6)
p53 overexpression,	
Absent	16 (44.4)
Present	20 (55.6)
Basal phenotype	
Absent	6 (16.7)
Present	30 (83.3)

TNBC, triple-negative breast cancer; DCIS, ductal carcinoma in situ;

Table S2. Clinicopathological characteristics of 155 TNBC patients in the validation set

Characteristics	No (%)
Age at primary diagnosis, years	
<50	84 (54.2)
≥50	71 (45.8)
Sex	
Female	155 (100.0)
Male	0 (0.0)
Histologic type	
Invasive carcinoma of no special type	142 (91.6)
Carcinoma with apocrine differentiation	12 (7.7)
Metaplastic carcinoma	1 (0.6)
T stage	
1	63 (40.6)
2	85 (54.8)
3	6 (3.9)
4	1 (0.6)
N stage	
0	116 (74.8)
1	24 (15.5)
2	8 (5.2)
3	7 (4.5)
M stage	
0	155 (100.0)
1	0 (0.0)
AJCC stage	
I	52 (33.5)
II	88 (56.8)
III	15 (9.7)
IV	0 (0.0)
Lymphovascular invasion	
Absent	99 (63.9)
Present	56 (36.1)
Histologic grade	
I	0 (0.0)
II	13 (8.4)
III	142 (91.6)
Nuclear pleomorphism	
1	0 (0.0)
2	11 (7.1)
3	144 (92.9)
DCIS component	
Absent	46 (29.7)
Present	109 (70.3)
Tumor border	
Pushing	95 (61.3)
Infiltrative	60 (38.7)
Multicentricity	
Absent	130 (83.9)
Present	25 (16.1)
Tumor-infiltrating lymphocyte	
<10%	48 (31.0)
≥10% and <50%	58 (37.4)
≥50%	49 (31.6)
Ki-67 index	
<50%	82 (52.9)

≥50%	73 (47.1)
p53 overexpression,	
Absent	69 (44.5)
Present	86 (55.5)
Basal phenotype	
Absent	23 (14.8)
Present	132 (85.2)

TNBC, triple-negative breast cancer; DCIS, ductal carcinoma in situ

Table S3. Correlation between tumor-infiltrating lymphocyte and its subsets

Variables	TIL	CD4⁺ TIL	CD8⁺ TIL	CD56⁺ TIL	CD57⁺ TIL	GNLY⁺ TIL	GZMB⁺ TIL
TIL	1						
$CD4^+$ TIL	0.589 (<0.001)	1					
$CD8^+$ TIL	0.557 (<0.001)	0.806 (<0.001)	1				
$CD56^+$ TIL	0.565 (<0.001)	0.625 (<0.001)	0.535 (<0.001)	1			
$CD57^+$ TIL	0.631 (<0.001)	0.671 (<0.001)	0.671 (<0.001)	0.589 (<0.001)	1		
$GNLY^+$ TIL	0.718 (<0.001)	0.690 (<0.001)	0.621 (<0.001)	0.697 (<0.001)	0.719 (<0.001)	1	
$GZMB^+$ TIL	0.582 (<0.001)	0.551 (<0.001)	0.553 (<0.001)	0.534 (<0.001)	0.621 (<0.001)	0.740 (<0.001)	1

Rho correlation coefficients calculated by Spearman′s rank correlation test are presented with *p* values in parentheses.

TIL, tumor-infiltrating lymphocyte; GNLY, granulysin; GZMB, granzyme B

Table S4. Correlation between tumor-infiltrating lymphocyte and its subset ratios

Variables	TIL	$CD4^+/CD8^+$	$CD56^+/CD8^+$	$CD57^+/CD8^+$	$GNLY^+/CD8^+$	$GZMB^+/CD8^+$	$CD56^+/CD4^+$	$CD57^+/CD4^+$	$GNLY^+/CD4^+$	$GZMB^+/CD4^+$
TIL	1									
$CD4^+/CD8^+$	0.158 (0.051)	1								
$CD56^+/CD8^+$	0.413 (<0.001)	0.295 (<0.001)	1							
$CD57^+/CD8^+$	0.387 (<0.001)	0.242 (0.003)	0.300 (<0.001)	1						
$GNLY^+/CD8^+$	0.528 (<0.001)	0.386 (<0.001)	0.477 (<0.001)	0.507 (<0.001)	1					
$GZMB^+/CD8^+$	0.434 (<0.001)	0.115 (0.156)	0.339 (<0.001)	0.336 (<0.001)	0.558 (<0.001)	1				
$CD56^+/CD4^+$	0.394 (<0.001)	0.020 (0.810)	0.924 (<0.001)	0.218 (0.007)	0.387 (<0.001)	0.297 (<0.001)	1			
$CD57^+/CD4^+$	0.351 (<0.001)	-0.255 (0.002)	0.115 (0.162)	0.817 (<0.001)	0.358 (<0.001)	0.272 (0.001)	0.234 (0.004)	1		
$GNLY^+/CD4^+$	0.474 (<0.001)	-0.151 (0.066)	0.351 (<0.001)	0.407 (<0.001)	0.830 (<0.001)	0.505 (<0.001)	0.431 (<0.001)	0.545 (<0.001)	1	
$GZMB^+/CD4^+$	0.377 (<0.001)	-0.156 (0.057)	0.254 (0.002)	0.309 (<0.001)	0.460 (<0.001)	0.953 (<0.001)	0.269 (0.001)	0.342 (<0.001)	0.524 (<0.001)	1

Rho correlation coefficients calculated by Spearman's rank correlation test are presented with *p* values in parentheses.
TIL, tumor-infiltrating lymphocyte; GNLY, granulysin; GZMB, granzyme B

Table S5. Relationships between TIL levels and clinicopathological features of tumors

**Clinicopathological features**		**Low-TIL group (*n*=48)**	**High-TIL group (*n*=107)**	***p* value**
Age (years)				0.293
	<50	23 (47.9)	61 (57.0)	
	≥50	25 (52.1)	46 (43.0)	
Histologic type				0.755
	IC-NST	45 (93.8)	97 (90.7)	
	Others[a]	3 (6.3)	10 (9.3)	
T stage				0.030
	T1-2	43 (89.6)	105 (98.1)	
	T3-4	5 (10.4)	2 (1.9)	
N stage				0.116
	N0	32 (66.7)	84 (78.5)	
	N1-3	16 (33.3)	23 (21.5)	
AJCC stage				0.382
	I	15 (31.3)	37 (34.6)	
	II	26 (54.2)	62 (57.9)	
	III	7 (14.6)	8 (7.5)	
Lymphovascular invasion				0.006
	Absent	23 (47.9)	76 (71.0)	
	Present	25 (52.1)	31 (29.0)	
Histologic grade				0.112
	II	7 (14.6)	6 (5.6)	
	III	41 (85.4)	101 (94.4)	
Nuclear pleomorphism				0.036
	2	7 (14.6)	4 (3.7)	
	3	41 (85.4)	103 (96.3)	
DCIS component				0.006
	Absent	7 (14.6)	39 (36.4)	
	Present	41 (85.4)	68 (63.6)	
Tumor border				0.022
	Pushing	23 (47.9)	72 (67.3)	
	Infiltrative	25 (52.1)	35 (32.7)	
Multicentricity				0.552
	Absent	39 (81.3)	91 (85.0)	
	Present	9 (18.8)	16 (15.0)	
Ki-67 index				0.051
	<50%	31 (64.6)	51 (47.7)	
	≥50%	17 (35.4)	56 (52.3)	
p53 overexpression				0.568
	Absent	23 (47.9)	46 (43.0)	
	Present	25 (52.1)	61 (57.0)	
Basal phenotype				0.668
	Absent	8 (16.7)	15 (14.0)	
	Present	40 (83.3)	92 (86.0)	

[a]Include metaplastic carcinoma and carcinoma with apocrine differentiation

Numbers in parentheses indicate column percentage.

TIL, tumor-infiltrating lymphocyte; IC-NST, invasive carcinoma of no special type; AJCC, American Joint Committee on Cancer; DCIS, ductal carcinoma in situ

Table S6. Relationship between clinicopathological characteristics and TIL subset ratios using $CD8^+$ TIL

Characteristics	$CD4^+/CD8^+$ TIL		$CD56^+/CD8^+$ TIL		$CD57^+/CD8^+$ TIL		$GNLY^+/CD8^+$ TIL		$GZMB^+/CD8^+$ TIL	
	ratio	*p value*	ratio	*p value*	ratio	*p value*	ratio	*p value*	ratio	*p value*
Age (year)		0.006		0.031		0.562		0.063		0.225
<50	1.10 (0.73-1.53)		0.01 (0.00-0.02)		0.03 (0.01-0.06)		0.07 (0.03-0.17)		0.05 (0.01-0.18)	
≥50	0.83 (0.52-1.21)		0.00 (0.00-0.02)		0.03 (0.01-0.05)		0.05 (0.01-0.14)		0.03 (0.01-0.10)	
Histologic type		0.363		0.704		0.543		0.471		0.573
IC-NST	0.94 (0.63-1.50)		0.01 (0.00-0.02)		0.03 (0.01-0.06)		0.06 (0.02-0.15)		0.04 (0.01-0.14)	
Others[a]	0.87 (0.53-1.17)		0.01 (0.00-0.04)		0.03 (0.01-0.04)		0.04 (0.01-0.16)		0.02 (0.01-0.12)	
T stage		0.209		0.662		0.819		0.034		0.126
T1-2	0.94 (0.63-1.47)		0.01 (0.00-0.02)		0.03 (0.01-0.06)		0.06 (0.02-0.16)		0.04 (0.01-0.15)	
T3-4	0.80 (0.14-1.06)		0.00 (0.00-0.04)		0.03 (0.01-0.13)		0.03 (0.00-0.05)		0.01 (0.00-0.06)	
N stage		0.738		0.422		0.475		0.153		0.133
N0	0.91 (0.61-1.35)		0.01 (0.00-0.02)		0.03 (0.01-0.06)		0.06 (0.03-0.17)		0.05 (0-0.14)	
N1-3	1.00 (0.64-1.61)		0.01 (0.00-0.02)		0.03 (0.01-0.05)		0.05 (0.01-0.12)		0.02 (0-0.15)	
AJCC stage		0.115		0.169		0.195		0.246		0.065
I-II	0.94 (0.63-1.48)		0.01 (0.00-0.02)		0.03 (0.01-0.06)		0.06 (0.02-0.16)		0.04 (0.01-0.15)	
III	0.77 (0.34-1.13)		0.00 (0.00-0.02)		0.02 (0.01-0.04)		0.04 (0.03-0.07)		0.01 (0.00-0.05)	
LVI		0.611		0.004		0.002		0.008		0.116
Absent	0.95 (0.61-1.39)		0.01 (0.00-0.03)		0.04 (0.01-0.07)		0.08 (0.03-0.18)		0.04 (0.01-0.16)	
Present	0.91 (0.63-1.51)		0.00 (0.00-0.02)		0.02 (0.01-0.04)		0.05 (0.01-0.08)		0.02 (0.00-0.12)	
Histologic grade		0.624		0.069		0.092		0.003		0.070
II	0.94 (0.53-1.47)		0.00 (0.00-0.01)		0.02 (0.00-0.04)		0.02 (0.00-0.05)		0.01 (0.00-0.08)	
III	0.92 (0.64-1.42)		0.01 (0.00-0.02)		0.03 (0.01-0.06)		0.07 (0.03-0.17)		0.04 (0.01-0.15)	
Nuclear pleomorphism		0.631		0.561		0.811		0.022		0.482
2	1.20 (0.51-1.41)		0.00 (0.00-0.03)		0.03 (0.00-0.07)		0.01 (0.00-0.06)		0.01 (0.00-0.16)	
3	0.92 (0.64-1.44)		0.01 (0.00-0.02)		0.03 (0.01-0.06)		0.06 (0.03-0.16)		0.04 (0.01-0.14)	
DCIS component		0.517		0.006		0.034		0.009		0.073
Absent	0.99 (0.65-1.41)		0.02 (0.00-0.03)		0.04 (0.02-0.09)		0.10 (0.03-0.20)		0.07 (0.01-0.16)	
Present	0.91 (0.61-1.47)		0.01 (0.00-0.02)		0.03 (0.01-0.05)		0.06 (0.02-0.13)		0.03 (0.00-0.14)	
Tumor border		0.603		0.102		0.053		<0.001		0.003
Pushing	0.94 (0.64-1.37)		0.01 (0.00-0.03)		0.04 (0.01-0.06)		0.09 (0.03-0.20)		0.06 (0.01-0.16)	
Infiltrative	0.88 (0.54-1.53)		0.01 (0.00-0.02)		0.03 (0.01-0.05)		0.04 (0.01-0.07)		0.01 (0.00-0.07)	
Multicentricity		0.969		0.869		0.274		0.765		0.646
Absent	0.92 (0.63-1.42)		0.01 (0.00-0.02)		0.03 (0.01-0.06)		0.06 (0.02-0.16)		0.03 (0.01-0.14)	
Present	0.94 (0.60-1.49)		0.01 (0.00-0.02)		0.02 (0.00-0.05)		0.06 (0.02-0.13)		0.04 (0.00-0.15)	
p53 overexpression		0.608		0.744		0.490		0.297		0.922
Absent	0.89 (0.64-1.34)		0.01 (0.00-0.02)		0.03 (0.01-0.05)		0.06 (0.02-0.13)		0.04 (0.01-0.14)	
Present	0.96 (0.61-1.55)		0.01 (0.00-0.02)		0.03 (0.01-0.06)		0.06 (0.03-0.18)		0.03 (0.01-0.16)	
Ki-67 index		0.578		0.025		0.294		0.006		0.128
<50%	0.93 (0.56-1.52)		0.01 (0.00-0.02)		0.03 (0.01-0.06)		0.05 (0.01-0.14)		0.02 (0.00-0.14)	
≥50%	0.93 (0.65-1.37)		0.01 (0.00-0.02)		0.03 (0.02-0.06)		0.09 (0.04-0.18)		0.05 (0.01-0.16)	
Basal phenotype		0.051		0.209		0.913		0.972		0.631
Absent	0.76 (0.48-1.10)		0.01 (0.00-0.03)		0.04 (0.00-0.06)		0.06 (0.01-0.22)		0.03 (0.00-0.15)	
Present	0.96 (0.64-1.52)		0.01 (0.00-0.02)		0.03 (0.01-0.06)		0.06 (0.03-0.14)		0.04 (0.01-0.14)	

Data are presented as median (interquartile range), and p values were calculated by Mann-Whitney U test.
[a]Includes metaplastic carcinoma and carcinoma with apocrine differentiation
TIL, tumor-infiltrating lymphocyte; GNLY, granulysin; GZMB, granzyme B; IC-NST, invasive carcinoma of no special type; AJCC, American Joint Committee on Cancer; LVI, lymphovascular invasion; DCIS, ductal carcinoma in situ

Table S7. Relationship between clinicopathological characteristics and TIL subset ratios using $CD4^+$ TIL

Characteristics	$CD56^+/CD4^+$ TIL		$CD57^+/CD4^+$ TIL		$GNLY^+/CD4^+$ TIL		$GZMB^+/CD4^+$ TIL	
	ratio	*p value*	ratio	*p value*	ratio	*p value*	ratio	*p value*
Age (year)		0.039		0.656		0.415		0.907
<50	0.01 (0.00-0.02)		0.03 (0.01-0.06)		0.07 (0.03-0.17)		0.04 (0.01-0.16)	
≥50	0.01 (0.00-0.02)		0.03 (0.01-0.08)		0.06 (0.02-0.13)		0.04 (0.01-0.11)	
Histologic type		0.840		0.415		0.925		0.912
IC-NST	0.01 (0.00-0.02)		0.03 (0.01-0.07)		0.06 (0.03-0.14)		0.04 (0.01-0.15)	
Others[a]	0.01 (0.00-0.03)		0.03 (0.01-0.05)		0.09 (0.01-0.23)		0.02 (0.01-0.16)	
T stage		0.214		0.702		0.115		0.275
T1-2	0.01 (0.00-0.02)		0.03 (0.01-0.06)		0.06 (0.03-0.14)		0.04 (0.01-0.15)	
T3-4	0.00 (0.00-0.02)		0.03 (0.00-0.10)		0.04 (0.01-0.06)		0.01 (0.00-0.10)	
N stage		0.208		0.158		0.068		0.077
N0	0.01 (0.00-0.02)		0.04 (0.01-0.07)		0.07 (0.03-0.18)		0.04 (0.01-0.16)	
N1-3	0.01 (0.00-0.01)		0.03 (0.01-0.05)		0.04 (0.03-0.12)		0.02 (0.00-0.11)	
AJCC stage		0.248		0.397		0.878		0.085
I-II	0.01 (0.00-0.02)		0.03 (0.01-0.06)		0.06 (0.03-0.14)		0.04 (0.01-0.16)	
III	0.00 (0.00-0.02)		0.02 (0.01-0.05)		0.07 (0.03-0.14)		0.01 (0.00-0.05)	
LVI		0.002		0.005		0.013		0.073
Absent	0.01 (0.00-0.03)		0.04 (0.02-0.07)		0.08 (0.03-0.18)		0.04 (0.01-0.17)	
Present	0.01 (0.00-0.01)		0.02 (0.01-0.05)		0.04 (0.02-0.10)		0.02 (0.00-0.09)	
Histologic grade		0.106		0.035		0.001		0.051
II	0.00 (0.00-0.01)		0.02 (0.00-0.03)		0.02 (0.01-0.05)		0.01 (0.00-0.08)	
III	0.01 (0.00-0.02)		0.04 (0.01-0.07)		0.07 (0.03-0.15)		0.04 (0.01-0.16)	
Nuclear pleomorphism		0.571		0.512		0.007		0.319
2	0.00 (0.00-0.02)		0.03 (0.01-0.06)		0.01 (0.00-0.06)		0.01 (0.00-0.13)	
3	0.01 (0.00-0.02)		0.03 (0.01-0.06)		0.07 (0.03-0.14)		0.04 (0.01-0.15)	
DCIS component		0.004		0.097		0.008		0.047
Absent	0.01 (0.01-0.03)		0.04 (0.01-0.07)		0.09 (0.04-0.22)		0.06 (0.02-0.18)	
Present	0.01 (0.00-0.02)		0.03 (0.01-0.06)		0.05 (0.02-0.12)		0.03 (0.01-0.13)	
Tumor border		0.052		0.055		<0.001		0.003
Pushing	0.01 (0.00-0.03)		0.04 (0.02-0.07)		0.09 (0.04-0.20)		0.06 (0.02-0.17)	
Infiltrative	0.01 (0.00-0.02)		0.03 (0.01-0.05)		0.04 (0.02-0.09)		0.02 (0.00-0.08)	
Multicentricity		0.553		0.154		0.596		0.502
Absent	0.01 (0.00-0.02)		0.03 (0.01-0.07)		0.06 (0.03-0.14)		0.04 (0.01-0.15)	
Present	0.01 (0.00-0.01)		0.02 (0.01-0.05)		0.06 (0.02-0.15)		0.04 (0.00-0.12)	
p53 overexpression		0.540		0.309		0.392		0.869
Absent	0.01 (0.00-0.02)		0.03 (0.01-0.06)		0.06 (0.02-0.15)		0.04 (0.01-0.13)	
Present	0.01 (0.00-0.02)		0.03 (0.01-0.07)		0.07 (0.03-0.14)		0.03 (0.01-0.16)	
Ki-67 index		0.012		0.527		0.015		0.093
<50%	0.01 (0.00-0.02)		0.03 (0.01-0.07)		0.05 (0.02-0.13)		0.03 (0.01-0.13)	
≥50%	0.01 (0.00-0.03)		0.04 (0.02-0.06)		0.08 (0.04-0.20)		0.05 (0.01-0.16)	
Basal phenotype		0.061		0.753		0.282		0.728
Absent	0.02 (0.00-0.03)		0.03 (0.01-0.08)		0.10 (0.02-0.23)		0.05 (0.01-0.15)	
Present	0.01 (0.00-0.02)		0.03 (0.01-0.06)		0.06 (0.03-0.13)		0.04 (0.01-0.15)	

Data are presented as median (interquartile range), and *p* values were calculated by Mann-Whitney U test.
[a]Includes metaplastic carcinoma and carcinoma with apocrine differentiation
TIL, tumor-infiltrating lymphocyte; GNLY, granulysin; GZMB, granzyme B; IC-NST, invasive carcinoma of no special type; AJCC, American Joint Committee on Cancer; LVI, lymphovascular invasion; DCIS, ductal carcinoma in situ

Table S8. Distribution of tumor-infiltrating lymphocyte and its subsets

Distribution of TIL and its subsets	No (%)
TIL	
High in all slides	22 (14.2)
Heterogeneous	50 (32.3)
Low in all slides	83 (53.5)
$CD4^+$ TIL	
Low in all cores	51 (32.9)
Heterogeneous	74 (47.7)
High in all cores	25 (16.1)
Inconclusive	5 (3.2)
$CD8^+$ TIL	
Low in all cores	44 (28.4)
Heterogeneous	67 (43.2)
High in all cores	35 (22.6)
Inconclusive	9 (5.8)
$CD56^+$ TIL	
Low in all cores	53 (34.2)
Heterogeneous	57 (36.8)
High in all cores	38 (24.5)
Inconclusive	7 (4.5)
$CD57^+$ TIL	
Low in all cores	51 (32.9)
Heterogeneous	69 (44.5)
High in all cores	28 (18.1)
Inconclusive	7 (4.5)
$GNLY^+$ TIL	
Low in all cores	45 (29.0)
Heterogeneous	65 (41.9)
High in all cores	38 (24.5)
Inconclusive	7 (4.5)
$GZMB^+$ TIL	
Low in all cores	63 (40.6)
Heterogeneous	57 (36.8)
High in all cores	31 (20.0)
Inconclusive	4 (2.6)

TIL, tumor-infiltrating lymphocyte; GNLY, granulysin; GZMB, granzyme B
