# Supplementary figures and images for: Characterization of tumor-infiltrating lymphocytes and their spatial distribution in triple-negative breast cancer
Source: Breast Cancer Res. 2024 Dec 6;26:180. doi: 10.1186/s13058-024-01932-4 (PMC11622547; doi:10.1186/s13058-024-01932-4)

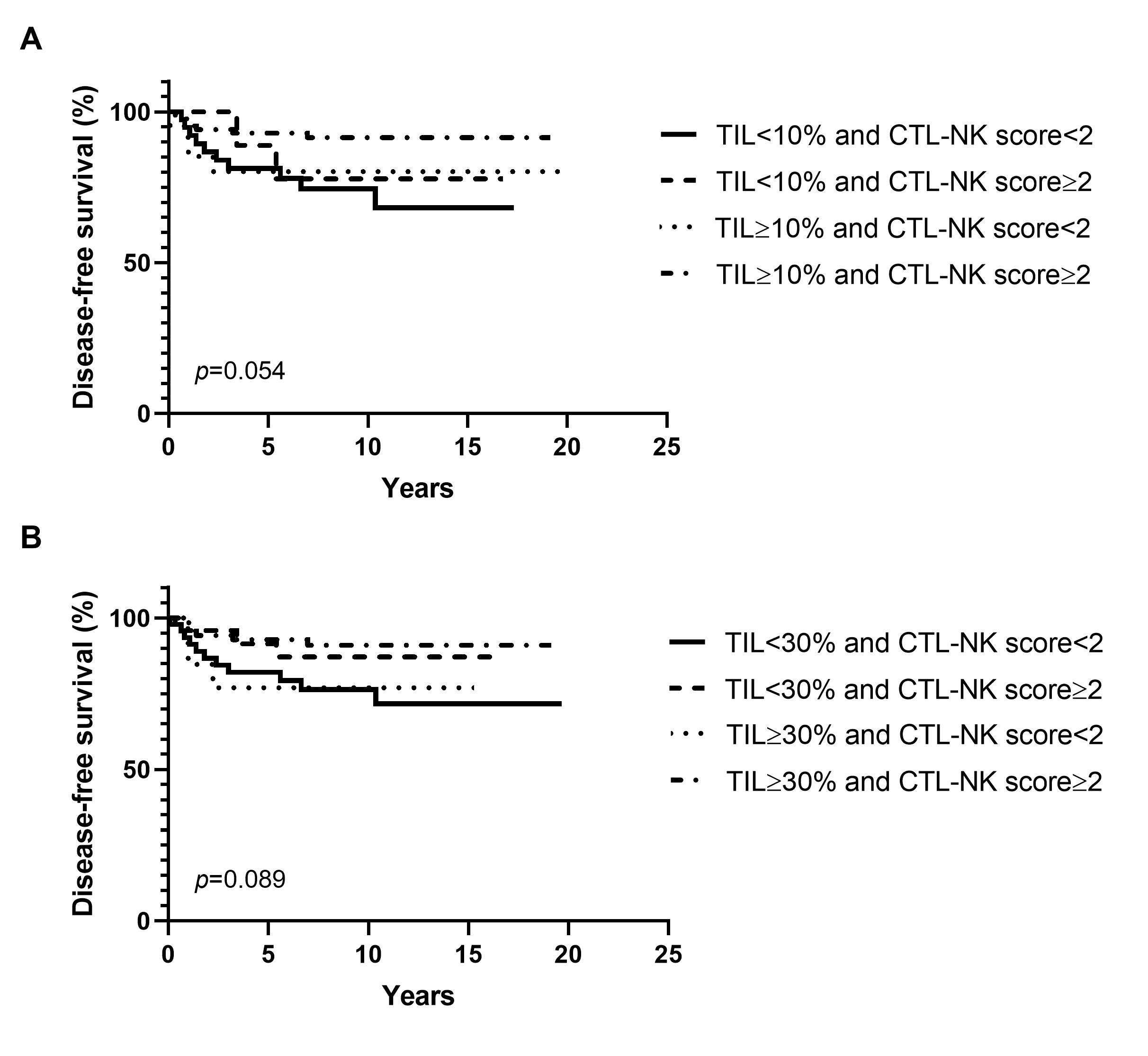

Supplement: Supplementary file 2 — Supplementary Material 2: Additional file 8: Figure S1. Kaplan-Meier survival curves for disease-free survival according to combined analyses of TIL levels (A, < 10% or ≥ 10%; B, < 30% or ≥ 30%) and CTL-NK scores (< 2 or ≥ 2). [file 13058_2024_1932_MOESM2_ESM.jpg]
